# Effects of an educational playful intervention on nasal hygiene behaviors of preschoolers: a quasi-experimental study

**DOI:** 10.15171/hpp.2019.06

**Published:** 2019-01-23

**Authors:** Priscila Costa, Talita Ermini, Cecília Helena de Siqueira Sigaud

**Affiliations:** ^1^Department of Pediatric Nursing, School of Nursing, Federal University of Sao Paulo, Sao Paulo, SP, Brazil; ^2^School of Nursing, Federal University of Sao Paulo, Sao Paulo, SP, Brazil; ^3^Department of Maternal-infant and Psychiatric Nursing, School of Nursing, University of Sao Paulo, Sao Paulo, SP, Brazil

**Keywords:** Child Day Care centers, Child health, Health education, Pediatric nursing

## Abstract

**Background:** To determine the effects of an educational playful intervention on nasal hygiene behaviors among preschool (3-4 years old) children.

**Methods:** A quasi-experimental before-after study was conducted with 39 children attending public daycare center in Sao Paulo, Brazil. A group-based intervention consisted of two educational sessions to promote healthy behaviors for nasal hygiene. It was adopted playful strategies such as a story told by puppets, card games and simulation of nasal hygiene in front of the mirror. The outcome was evaluated by observing six healthy behaviors for nasal hygiene one week before and after the intervention. Differences in the outcome before and after the intervention were tested using Wilcoxon signed rank test and McNemar’s test.

**Results: ** The median of healthy nasal hygiene behaviors went from 3.0 to 4.0 after the intervention, with a significant statistical difference (P=0.0004) and a difference of behaviors. After the intervention, forcing the air out of one opened nostril increased from 5.1%to 30.8% (P=0.001, CI 95%: 0-0.440), forcing the air out of the other nostril increased from 5.1% to 28.2% (P=0.003/CI 95%: 0-0.50), throwing the piece of toilet paper in the garbage increased from 53.8% to 87.21% (P=0.04, CI 95%: 0.035-0.65), and sanitizing the hands with soap and water increased from 15.4% to 43.6% (P=0.039-0.76).

**Conclusion: ** the educational playful intervention improved the autonomy of preschoolers to adopt healthy nasal hygiene behaviors.

## Introduction

Early childhood is the period of life when the architecture of the developing brain is most open to the influences of relationships and experiences, establishing either a sturdy or a weak foundation for health, learning, and behavior throughout life.^1^ Health education should promote equitable access to health promotion practices especially for young children living in low and middle-income communities.

Societal changes to mothers’ workforce participation have increased the relevance of daycare centers as a location to promote child’s health. Daycare centers have a pivotal role in shaping children’s healthy habits by providing the contextual environment and responsive care according to the needs of children under 4 years old. Globally, a considerable number of under-three children in modern society attend daycare. In the United States, almost 8 million children aged 3-4 years were at daycare centers in 2016 and most of them (55.5%) spending more than 30h per week.^2^ In Brazil, more than 3 million children under 4 years attended daycare centers in 2017 and 65% of them were at public institutions.^3^

Even considering the benefits of early childhood education for human development, attending daycare centers represents a risk factor for respiratory tract infection. The findings of a cohort study conducted with 1827 children from 1797 families showed the rapid increase in respiratory infections at 2 months after the start of daycare with a subsequent decrease within the following 9 months.^4^ A study that analyzed a web-based biosurveillance program in 4 childcare centers in a single Michigan county found that 46% of the 385 individual episodes of fever, gastroenteritis, influenza like illness, cold, rash, conjunctivitis, ear infection occurred with preschoolers.^5^ Therefore, interventions to promote healthy behaviors among children and staff of childcare educational settings in order to decrease the incidence of infectious diseases, such as respiratory infection and diarrhea,^6^ remains needed.

Considering preschoolers, playing is vital to develop the potential of every child during health education practices. Play helps children develop physically, mentally, emotionally and socially.^7^ Play allows children to use their creativity while developing their imagination, dexterity, physical, cognitive, and emotional skills.^8^ It is through play that children at a very early age engage, interact and master the world around them.^8^ As play offers an ideal opportunity for child development, healthcare professionals should adopt playful strategies in order to promote healthy behaviors and life skills for preschoolers.

Considering the need for health education practices in educational settings, previous studies investigated the effectiveness of interventions to promote oral health,^9^ healthy eating behaviors^10^ and hand washing^11^ in preschool children. However, evidence regarding the effects of an educational playful intervention on healthy nasal hygiene behaviors in children aged 3-4 years attending a daycare center remains unmet. Therefore, the aim of this study was to determine the effects of an educational playful intervention on nasal hygiene behaviors among preschool (3-4 years old) children.

## Materials and Methods

### Study design and setting 

This quasi-experimental, before-after study, was carried out at a public daycare center in Sao Paulo, Brazil. The daycare center provides full-time care for 200 children aged 0-4 years from a community with a high level of social vulnerability and low-income families. Data collection occurred from March to June 2017.

### Participants

To calculate the sample size, an α of 0.05 and β of 0.80 was assumed, in addition to a minimum difference of two healthy behaviors before and after the intervention, resulting in a minimum sample of 32 subjects. The participants included children aged between 3-4 years, willing to participate in the group-based educational playful intervention. Considering the possibility of losses during the intervention, the researchers chose to invite 64 children who met the inclusion criteria. The exclusion criteria were not participating of two group-based sessions that occurred once a week, impossibility to evaluate the outcome before and after the intervention, and having a suspected or diagnosed mental disease, reported by the caregiver of the daycare center.

### Intervention protocol

Two 60-minutes group-based educational sessions about nasal hygiene behaviors were held. Two nurses, members of the research team, planned and performed the intervention with the support of the daycare center caregivers. The sessions occurred once a week inside the classrooms of the preschoolers. Each session included from 20 to 30 preschool children of two different groups.

In the first session, after presenting a story told by puppets regarding healthy behaviors for nasal hygiene when they had flu, children were encouraged to discuss their opinion regarding the importance of nasal hygiene. Subsequently, each child was invited to stand in front of the mirror and put a crepe paper with a greenish gelatinous near the nose. This strategy aimed to simulate the need for nasal hygiene. Then, the child was encouraged to simulate nasal hygiene, followed by hand sanitizing with soap and water. At the end of this session, the child received a sticker of a superhero.

In the second session, a card game with illustrations of six healthy behaviors for nasal hygiene was presented. Children had to put the cards in the correct order according to the six steps of nasal hygiene. After that, each child was invited to perform nasal hygiene in front of a mirror and sanitize the hands with soap and water. Finally, children received a reward sticker with stars and phrases such as “well done”.

### Outcome measurement

The healthy behaviors related to nasal hygiene were verified in each child one week before and one week after the intervention. Based on literature,^12^ six healthy behaviors related to nasal hygiene were evaluated, as follows: to pick up a piece of toilet paper, to position it in the nose, to force the air out of one opened nostril, to force the air out of the other opened nostril, to throw the piece of toilet paper in the garbage, and to sanitize the hands in the sink with soap and water.

In order to evaluate the six behaviors related to nasal hygiene, the child was asked to demonstrate what she/he would do when the nose was in need to be cleaned. This evaluation was performed in the bathroom of the children´s classroom that was a familiar place to them. One of the authors observed the response of each child individually and checked the presence or absence of each one of the six behaviors mentioned above. This evaluation was registered on a data collection instrument that had the name of the child, date, and the six behaviors.

Overall children were comfortable demonstrating the behaviors adopted in order to perform nasal hygiene. Keeping the evaluation inside the bathroom of the children’s classroom and close to the educators, and adopting a gentle approach to each child facilitated that each child demonstrated behaviors related to nasal hygiene.

### Data analysis

Data were analyzed using the R Statistical software. The normality of the data was examined by the Shapiro-Wilk normality test. As our data were non-parametric, differences in the outcome before and after the intervention were tested using Wilcoxon signed rank test for quantitative data and McNemar’s test to analyze paired nominal data. The data were described as absolute and relative frequency, median, means, maximum and minimum values. All the results was considered as significant with *P* < 0.05.

## Results

**Figure 1:**
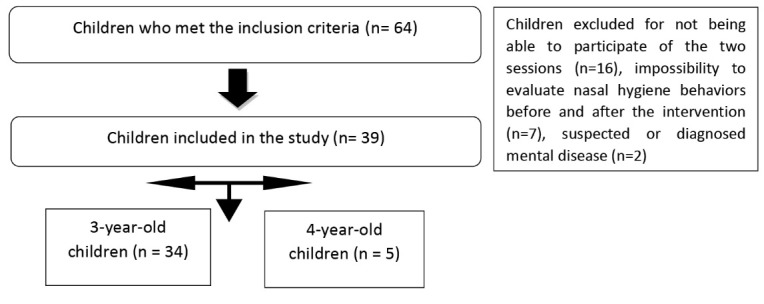
Participants of the study, São Paulo, Brazil, 2017.

A total of 39 children participated in the study, according to Figure 1. Most of them had 3 years of age (87.2%) and were female (64.1%).

Children were enthusiastic about participating in the intervention, facilitating their recruitment. During intervention sessions, it was observed that children were particularly interested and excited about having a story told by puppets, practicing nasal hygiene in front of the mirror using crepe paper with a greenish gelatinous near the nose and receiving a reward sticker at the end of each session. The use of playful activities was considered crucial in determining the success of these activities. We observed that 3-year-old children showed some difficulty to play with the card game and 4-year-old children were excited about this challenge.

There was an increased adoption of healthy nasal hygiene behaviors after the playful intervention with preschoolers (Table 1). The most common healthy behaviors before intervention were to pick up a piece of toilet paper and to position it in the nose. After the intervention, forcing the air out of one opened nostril increased from 5.1% to 30.8% (*P* = 0.001, CI 95%: 0-0.440), forcing the air out of the other nostril increased from 5.1% to 28.2% (*P* = 0.003, CI 95%: 0-0.50), throwing the piece of toilet paper in the garbage increased from 53.8% to 87.21% (*P* = 0.04, CI 95%: 0.035-0.65), and sanitizing the hands with soap and water increased from 15.4% to 43.6% (*P* = 0.039-0.76).

Findings showed that educational playful intervention had a significant effect on healthy behav­iors for nasal hygiene. The median number of healthy behaviors for nasal hygiene before the intervention was 3.0 ranging from zero to six. After the intervention, the median of healthy behaviors was 4.0, ranging from zero to six with a statically significant difference (*P* = 0.0004, CI 95%: 0.99-2.0), according to Table 2. The effects of a playful intervention about nasal hygiene were also analyzed according to the child’s age and sex.

Female, 3-year old and 4-year old children increased the number of healthy behaviors after the intervention.

## Discussion

Results of the study showed that the educational playful intervention considerably increased adoption of healthy behaviors for nasal hygiene among preschool children at a daycare center. Our findings suggested a positive effect of the intervention on six behaviors related to the child’s autonomy performing nasal hygiene. This study supports health education practices provided by nurses at daycare centers, in order to promote child’s health and to prevent the dissemination of respiratory diseases.

After intervention, there was an improvement of the six healthy behaviors for nasal hygiene. Picking up a piece of toilet paper increased 20.5%, positioning a piece of toilet paper in the nose increased 15.4%, forcing the air out of the opened nostril at a time increased more than 23%, throwing the piece of toilet paper in the garbage and sanitizing the hands in the sink with soap and water increased 33.4% and 28.2%, respectively.

The most frequent nasal hygiene behaviors that we found demonstrated the initiative of preschool children to care of themselves. Our findings showed that after the playful intervention, more than 80% of the preschoolers adopted three fundamental nasal hygiene behaviors that included picking up a piece of toilet paper, positioning it in the nose, and throwing it in the garbage.

Our results showed that children learned healthy nasal hygiene behaviors when supported by adults that adopted playful strategies for health education. Considering that play contributes to brain development, learning, physical and social development^13^ our intervention adopted playful strategies such as a story told by puppets, card games and simulation of nasal hygiene in front of the mirror. Likewise, authors^14^ of a review of literature have suggested that the use of puppets in health care is emerging as a mode of simulation that combines elements of engaging with the child through play whilst at the same time providing education.

Similarly, previous studies^9-11^ performing health education practices with preschool children required the adoption of playing strategies such as stories, painting, games, and puzzles in order to promote healthy behaviors related to personal hygiene. A study conducted with 78 preschool children (2-6 years old) adopted playful strategies such as games, cartoons, drawing, and interaction with an adult dressed as a superhero during 12 months in a Brazilian kindergarten found that 62.7% of children learned about how to prevent anemia and had healthier eating habits encouraged.^15^ As innovation is crucial to improving healthy behaviors, a study showed that preschool children can play and understand messages about diarrhea prevention using games as a learning device.^16^

During early childhood, health and education professionals play a vital role in promoting healthy behaviors in educational settings. A randomized controlled trial conducted in Italy with 425 3-year-old children at 16 childcare centers demonstrated the positive impact of a combined educational intervention carried out by primary care pediatricians and childcare center teachers. The intervention changed four energy-related behaviors in children: fruit and vegetable intake, physical activity, TV-watching time and sugar-sweetened beverages intake. After 2 years from baseline significant and beneficial changes in behaviors took place among those intervention children whose mothers had a medium/high level of education.^17^ Therefore interprofessional care provided by nurses and daycare caregivers, which represent a source of emotional support for children, has the potential to positively affect the care of preschool children in educational settings.

Health education interventions highlight that the role of healthcare professionals is evolving to focus on providing preventive and acute child care, but also place greater emphasis on the context of routine care of children at educational settings of the community. Neuroscience is also producing extensive evidence suggesting that the later we wait to support caregivers with children, the more difficult and likely more costly it will be to achieve positive outcomes.^1^ Early childhood investments should considerer prenatal-to-three period for families and caregivers taking care of children in a context of adversity and social vulnerability.^1^

This study innovates proposing the evaluation of an educational playful intervention on nasal hygiene behaviors among preschool children. Although we analyzed data from 39 preschool children, some limitations related to study design, unicentric setting, participants and outcome measurement should be considered. Regarding outcome measurement, a potential source of bias relates to the evaluation of each child by the same researchers who implemented the intervention. Our small sample, eligibility criteria and source of population considering a single daycare center compromises the power of the study and subsequently the external validity of our results. Recommendations for future studies include conducting randomized controlled trials with a larger sample size of multiple daycare centers, and also developing and validating outcome measurements tools to evaluate nasal hygiene behaviors among preschool children.

## Conclusion

The educational playful intervention improved the autonomy of preschoolers in order to adopt healthy nasal hygiene behaviors. Interprofessional care provided by health and education professionals are required to promote health and prevent diseases at daycare centers.

## Ethical approval

All procedures performed in the study involving human participants were in accordance with the ethical standards of the 1964 Helsinki declaration and its later amendments or comparable ethical standards. The research project was approved by the Research Ethics Committee of the School of Nursing at Federal University of São Paulo (Protocol 1.849.484). Child’s parents signed an informed consent form. Before initiating data collection, we explained the activity to each child and asked if she/he would like to participate. Children demonstrated joy and enthusiasm during playful intervention sessions.

## Competing interests

The authors declare that they have no competing interests.

## Funding

No fund was received to conduct this study.

## Authors’ contributions

PC was involved in the conception of the study, data collection, analysis and interpretation of the data and drafted the manuscript. TE contributed to the study design, data collection, analysis and interpretation, and critically revised the manuscript. CHSS contributed to the study design, oversaw the whole study process, conducted the interpretation of data, and revised the manuscript. All authors read and approved the final manuscript.

## Acknowledgments

We would like to thank educators, the direction board of the daycare center and also children and their families.

**Table 1:** Healthy behaviors for nasal hygiene before and after a playful intervention with preschool children. Sao Paulo, Brazil, 2017

**Healthy behaviors for nasal hygiene**	**Before intervention** **No. (%)**	**After intervention No. (%)**	**Difference between proportions**	**95% CI**	***P*** ** value**
Pick up a piece of toilet paper	28 (71.8)	36 (92.3)	20.5%	0.04-1.032	0.057
Position a piece of toilet paper in the nose	27 (69.2)	33 (84.6)	15.4%	0.15-1.43	0.23
Force the air out of one opened nostril	2 (5.1)	12 (30.8)	25.7%	0-0.44	0.0019
Force the air out of the other opened nostril	2 (5.1)	11 (28.2)	23.1%	0-0.5066	0.0039
Throw the piece of toilet paper in the garbage	21 (53.8)	34 (87.2)	33.4%	0.035-0.655	0.004
Sanitize the hands in the sink with soap and water	6 (15.4)	17 (43.6)	28.2%	0.039-0.7677	0.012

**Table 2:** Comparison of the number of healthy behaviors for nasal hygiene before and after a playful intervention. São Paulo, Brazil, 2017

	**Before intervention**	**After intervention**	**Median**	***P*** ** value**	**95% CI**
	**Mean (SD)**	**Mean (SD)**			
No. of healthy behaviors	2.3 (1.60)	3.66 (1.62)	1.99	0.0004	0.99-2.00
Sex					
Female	2.4 (1.68)	3.92 (1.63)	1.50	0.001	0.99-2.49
Male	2.1 (1.51)	3.21 (1.57)	1.60	0.12	0.49-3.0
Child age					
3-year old children	2.26 (1.63)	3.55 (1.67)	1.99	0.02	0.99-2.49
4-year old children	2.60 (1.51)	4.40 (1.14)	1.99	0.03	1.0-2.0
